# Transactions of the American Ophthalmological Society

**Published:** 1872-05

**Authors:** 


*Transactions of the American Ophthalmological Society.* Eighth Annual meeting. Newport, July, 1871. New York: D. APPLETON & Co. 1871.

The transactions of this Society are full of interesting and valuable matter. Dr. Jeffries gives an interesting and exhaustive report on the progress of Ophthalmology, in which he pays a short and deserved tribute to Von Graefe and other noted Ophthalmologists who have recently died. The whole Volume is full of interesting papers and shows that the society is fully awake to the importance of its mission.

